# Clinical Potential of a New Approach to MRI Acceleration

**DOI:** 10.1038/s41598-018-36802-5

**Published:** 2019-02-13

**Authors:** Nadine L. Dispenza, Sebastian Littin, Maxim Zaitsev, R. Todd Constable, Gigi Galiana

**Affiliations:** 1 Department of Biomedical Engineering, Yale University, New Haven, CT USA; 2 Department of Diagnostic Radiology, Medical Physics, University Medical Center Freiburg, Breisacher Str. 60a, 79106 Freiburg, Germany; 3 Department of Radiology and Biomedical Imaging, Yale University, New Haven, CT 06520 USA; 4 Department of Neurosurgery, Yale University, New Haven, CT 06520 USA

## Abstract

Fast ROtary Nonlinear Spatial ACquisition (FRONSAC) was recently introduced as a new strategy that applies nonlinear gradients as a small perturbation to improve image quality in highly undersampled MRI. In addition to experimentally showing the previously simulated improvement to image quality, this work introduces the insight that Cartesian-FRONSAC retains many desirable features of Cartesian imaging. Cartesian-FRONSAC preserves the existing linear gradient waveforms of the Cartesian sequence while adding oscillating nonlinear gradient waveforms. Experiments show that performance is essentially identical to Cartesian imaging in terms of (1) resilience to experimental imperfections, like timing errors or off-resonance spins, (2) accommodating scan geometry changes without the need for recalibration or additional field mapping, (3) contrast generation, as in turbo spin echo. Despite these similarities to Cartesian imaging, which provides poor parallel imaging performance, Cartesian-FRONSAC consistently shows reduced undersampling artifacts and better response to advanced reconstruction techniques. A final experiment shows that hardware requirements are also flexible. Cartesian-FRONSAC improves accelerated imaging while retaining the robustness and flexibility critical to real clinical use.

## Introduction

MRI is one of the safest and most informative imaging modalities available to modern medicine, but its overall applicability is limited by long imaging times. The bedrock of MRI is that evolution under a linear gradient allows one to sample the Fourier space (k-space) of an image *one point at a time*^1,2^. The first exception to this concept came in the early 90 s, when it was recognized that locally sensitive receiver coils sample a weighted collection of k-space points, creating potential for scan acceleration. The trajectory of the linear gradients translate that static sampling distribution to measure all of k-space. By using an array of such coils, one can solve for points of k-space that were only sampled by the wings of the sampling distributions, allowing some lines of data to be skipped in an acceleration approach known as parallel MRI.

The work presented here is based on regarding nonlinear gradients as the next step in this progression. Nonlinear gradient encoding also samples a distribution of k-space rather than a single point, whether used alone or in combination with receiver arrays. But with nonlinear gradients (NLGs), the sampling distribution can be updated dynamically during the readout of each line. (Supplementary Videos S1–S3)^3–6^.

The notion that NLGs could reduce MRI scan times was first hypothesized in the early 2000s, and this goal has since been pursued from many different angles. Since scan time can sometimes be limited by the switching time of the gradient field, several groups looked at using NLGs to reduce this time, either by reducing the number of switching events needed to encode an image^7^ or by switching more rapidly^8^. Another approach to reducing scan time is to image a smaller region, and several groups have shown that NLGs can be used to shrink the imaging region to some target in the anatomy^9–12^.

That nonlinear gradients could enhance parallel imaging by matching the spatial geometry of the gradients to that of receivers was also previously hypothesized and has been explored from several angles. O-space was the first imaging method explicitly designed to match receiver and gradient geometry, using a radially varying NLG to complement the azimuthal geometry of typical receiver arrays^13,14^. Many other schemes have since been proposed (NSI^15^, MDE^16^, 4D-RIO^17^), but among those that have been experimentally validated, the addition of NLG encoding has shown only moderate improvements over equivalent methods without NLG encoding^10,18–21^.

FRONSAC is a very different approach to matching receiver array and gradient shape for better undersampled imaging, based on complementarity in k-space rather than the spatial domain, where FRONSAC minimally changes the real encoded resolution of each voxel (Fig. S2)^22^. As previously mentioned, NLG encodings create a sampling distribution in k-space that can be varied dynamically as the linear gradients sweep the sampling distribution across k-space (Supplementary Videos S4 and S5)^23^. NLGs in conjunction with receiver arrays generate sets of dynamic sampling distributions that are simultaneously sampled at each timepoint. This additional degree of freedom can be used to design trajectories that more efficiently measure the gaps created by the “skipped” parts of k-space, which are sampled only by the wings of these distributions. In all cases, the linear gradients of a traditional trajectory translate these distributions across k-space until the entire space is sufficiently sampled.

**Figure 1:**
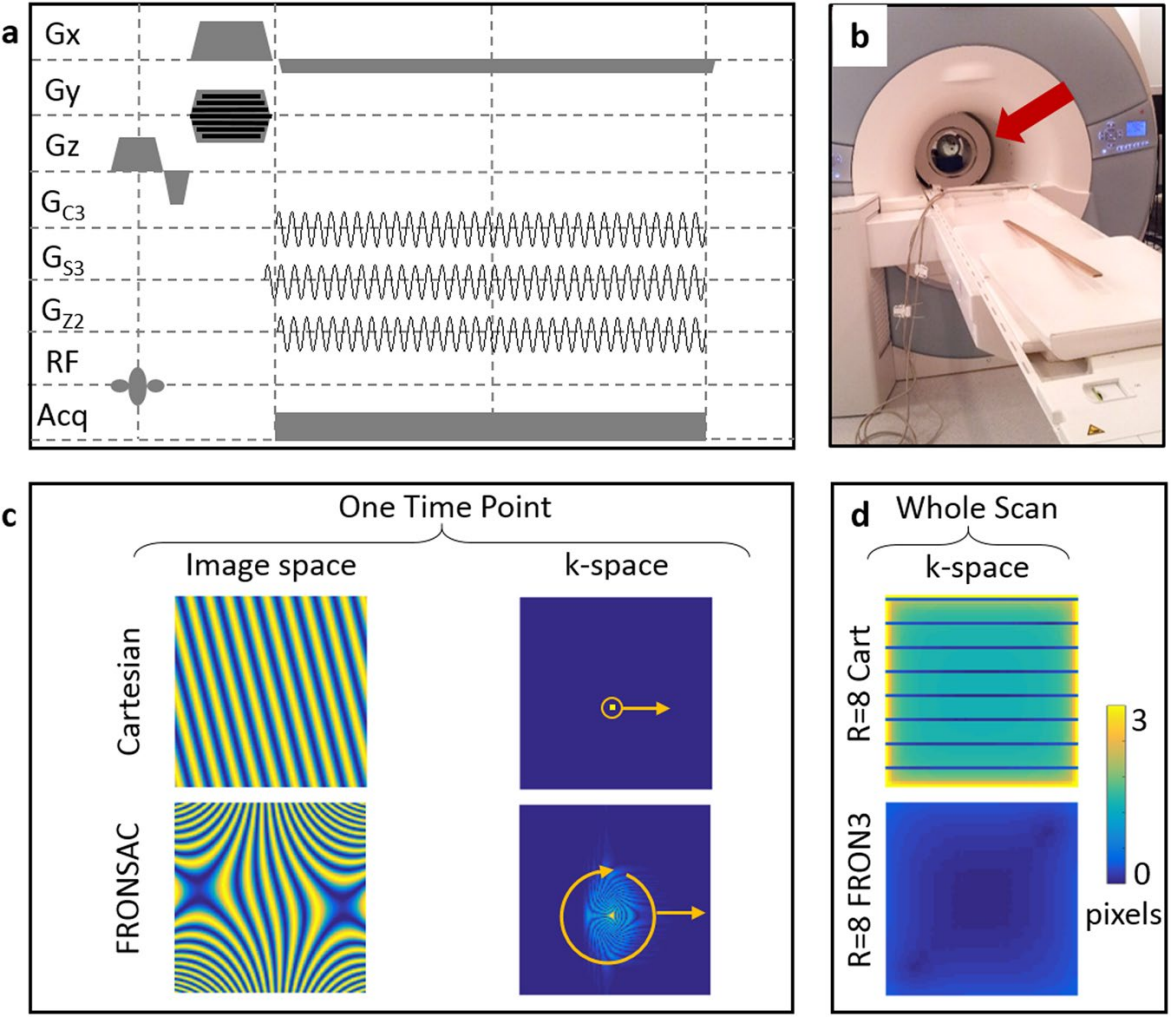
FRONSAC gradients improve sampling in gaps of k-space. (**a**) FRONSAC adds small, sinusoidal, nonlinear gradients to the readout of an existing pulse sequence. (**b**) The nonlinear gradient fields are generated by a coil within the magnet. (**c**) In the Cartesian case (top row) the linear modulation in image space corresponds to a sampling point translated through k-space. When FRONSAC gradients are added, the image space modulation is nonlinear, and it corresponds to sampling a rotating and translating distribution of k-space at each time point. Each of these are further modulated by coil encodings. (**d**) One way to measure k-space coverage is to calculate the width of a point spread function (PSF) in k-space reconstructed from the experimental encodings, including all timepoints and coils. An R = 8 Cartesian sequence with coil encoding does not fully solve for points in the gaps of k-space resulting in wide PSFs, whereas the addition of a FRONSAC 3 gradient, as further detailed in Fig. 2, greatly improves this sampling resulting in narrow PSFs.

One metric to evaluate the encoding efficiency of a NLG trajectory is the width of the PSF in k-space at each location when reconstructed from the acquired sampling distributions in k-space. Numerical optimization with this metric constrained by realistic hardware limitations yielded a sequence with highly dynamic NLG encoding similar to FRONSAC^24^. Figure 1d compares this metric evaluated for each point of k-space (the width of a sampling distribution reconstructed at each location in k-space) provided by an undersampled linear acquisition versus one enhanced by FRONSAC encoding. The addition of FRONSAC NLG encoding can improve image quality whether the nominal trajectory through k-space is rectilinear Cartesian or some arbitrary non-Cartesian path. It is a general approach to improve sampling of the gaps in any traditional linear trajectory through k-space. However, in this work we focus on improving undersampled Cartesian encoding, since it is nearly ubiquitous in clinical imaging due to its insensitivity to various experimental imperfections.

In this work we demonstrate that Cartesian FRONSAC, which is one particular instance of the FRONSAC approach where oscillating nonlinear gradients are added to a Cartesian sequence, not only provides excellent accelerated imaging, but it also exhibits a number of features critical to real world clinical imaging. Unlike many non-Cartesian trajectories known to improve parallel imaging, Cartesian-FRONSAC shows the same mild artifacts as standard Cartesian imaging in the presence of experimental imperfections, such as off-resonance spins or gradient errors. A single FRONSAC gradient enhances undersampled image quality for nearly any imaging prescription, despite changes in image dimension, orientation or resolution, so the method does not require extensive field mapping. It is applicable to a number of different sequence types, demonstrated here with gradient echo and fast spin echo sequences, yielding identical contrast. The experimental results also prove that FRONSAC imaging yields more benefit from the new generation of reconstruction algorithms which are now becoming commercially available^25–29^. Finally, we present data using a novel hardware setup with impure FRONSAC NLG encoding acquired at an entirely different laboratory, which shows that the method does not require spatially or temporally ideal gradients.

## Results

### Artifact mitigation with addition of non-optimized FRONSAC waveform sets

**Figure 2:**
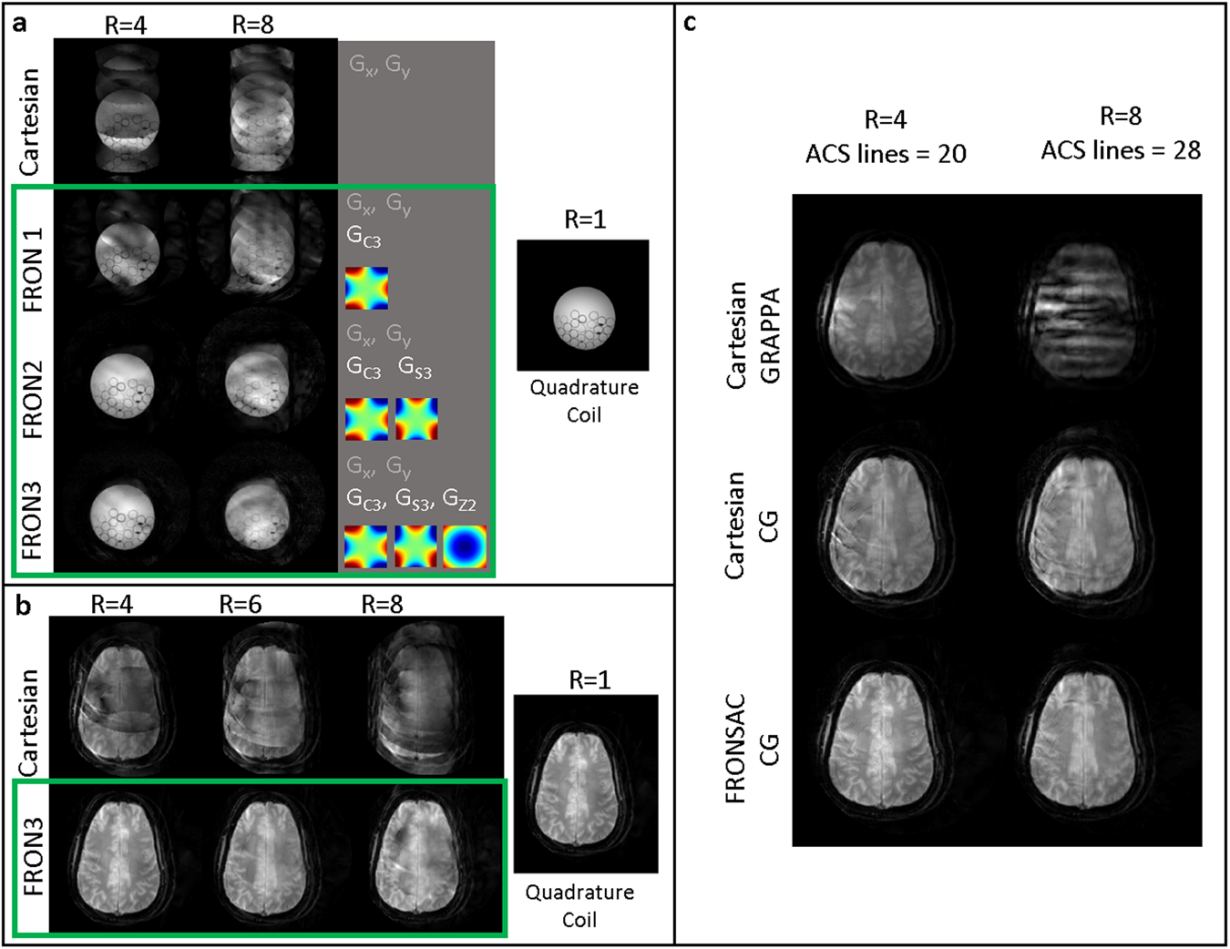
Folding artifacts due to undersampling improve with the addition of FRONSAC waveforms. (**a**) As the number of nonlinear gradient waveforms is increased from 1 to 3 (FRONSAC1 to FRONSAC3) the folding artifacts due to undersampling improve in phantom studies for undersampling factors of 4 and 8. Improvements in image quality are especially apparent with the out of phase application of two or more FRONSAC waveforms. The applied nonlinear gradient shapes, labeled with their common names, are shown as insets in the third column. (**b**) Using FRONSAC3 encoding, the results are validated *in vivo*. (**c**) FRONSAC image quality improvements are apparent whether comparing to Cartesian data reconstructed with GRAPPA or conjugate gradient.

Figure 2a shows experimental images where folding artifacts due to undersampling improve as a function of increasing number of NLG waveforms added to a Cartesian trajectory. Artifacts are mitigated from the addition of FRONSAC waveforms, despite the fact that these waveforms have not been optimized for frequency, phase, or amplitude and use only 10% of the available gradient amplitude. The rows compare images of a water bottle phantom reconstructed from undersampled Cartesian data (no NLG encoding) versus additional FRONSAC encoding with 1, 2, or 3 NLG waveforms. Scan times are reduced by acquiring fewer measurements to produce an image as shown in the columns with undersampling factors of 4 and 8. The applied field shapes are shown in the third column, each labelled with their common name. Defining equations for these shapes are available in Supplementary Note S1.

In each case, the FRONSAC encoding reduces the residual ghosting artifact due to undersampling, particularly in the presence of 2 or more waveforms, which provide more degrees of freedom to manipulate the sampling distribution. The 3 waveform case, chosen because it provides NLG encoding in all 3 dimensions, was also demonstrated *in vivo* (Fig. 2b), with no reported peripheral nerve stimulation.

In Cartesian brain images reconstructed with conjugate gradients (CG) or GRAPPA, ghosting in the anterior-posterior direction, a remnant of the undersampling in that direction, is greatly reduced by the additional nonlinear encoding (Fig. 2b,c). Since Fig. 2b shows all Cartesian images with CG reconstruction, there may be concerns that a GRAPPA reconstruction would reduce artifacts without the addition of nonlinear gradients. To address this, Fig. 2c shows that GRAPPA does not improve artifacts in this data, and the CG reconstruction does not give FRONSAC an unfair advantage over Cartesian acquisitions.

FRONSAC is a flexible approach that does not require predetermined optimized NLG waveforms.

### Performance in the presence of experimental imperfections

**Figure 3:**
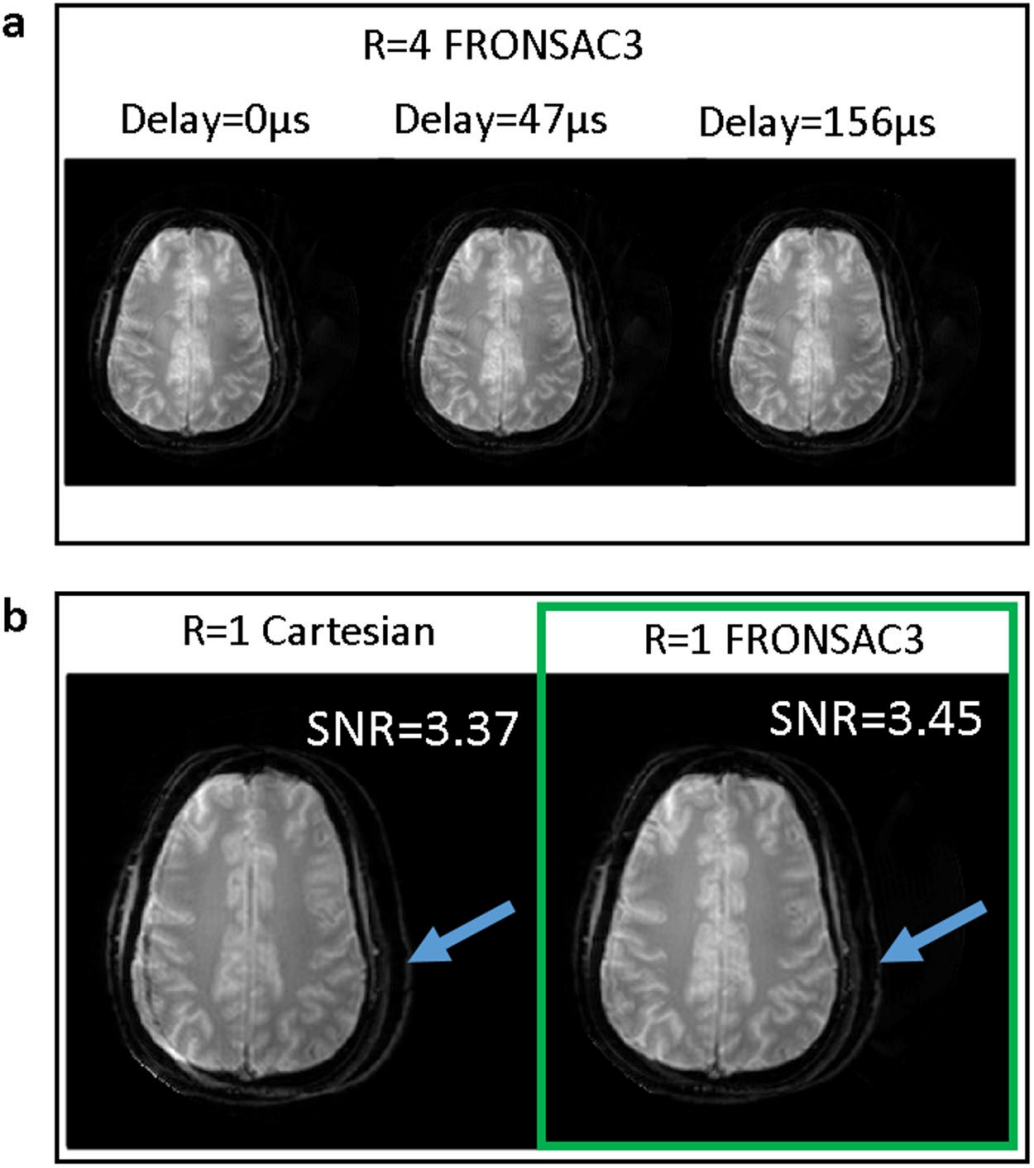
Because FRONSAC gradients provide a small perturbation to the encoding function, artifacts remain Cartesian-like and generally benign in the presence of experimental imperfections. For example, (**a**) image quality is not degraded by simulated timing delays in the linear gradients, and (**b**) off-resonance spins cause a simple shift in the readout direction, as seen in the shifted skull of this experimental brain image.

Figure 3 demonstrates that Cartesian-FRONSAC retains the same chemical shift artifact and off-resonance behavior as conventional Cartesian encoding, and the resulting artifacts are indistinguishable from well-known and generally benign Cartesian artifacts. Unlike many non-Cartesian trajectories that mitigate undersampling artifacts, Fig. 3a shows no degradation in the magnitude images for FRONSAC in the presence of a timing delay in the linear gradients, though the expected slope is generated in the phase. Note there is never a timing delay in the NLG gradient because it is empirically mapped and applied identically to each new scan. In addition, the off resonance artifact in Fig. 3b for the fat/water shift is identical in the low-bandwidth Cartesian and FRONSAC images; both images show a simple spatial shift with no further image distortion. This too is in contrast to many non-Cartesian trajectories that improve parallel imaging, which often give dramatic and complicated artifacts in the presence of off-resonance spins.

### Performance at various scan prescriptions using a single non-optimized FRONSAC waveform set

**Figure 4:**
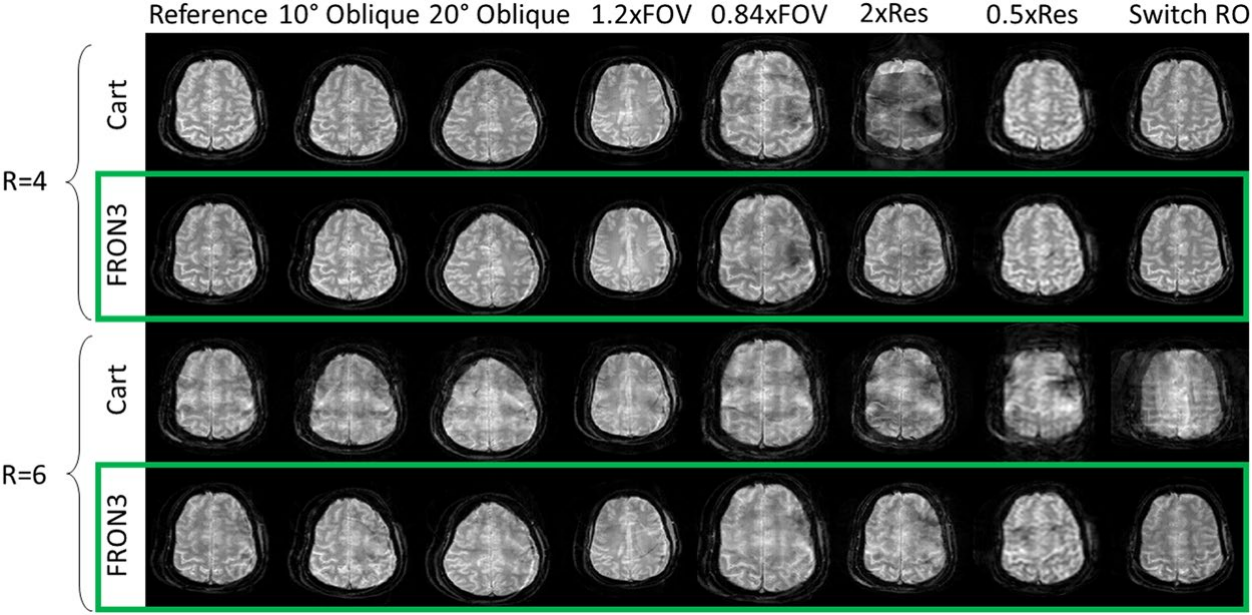
A single FRONSAC waveform improves many scan prescriptions. While routine changes in scan prescription (orientation, field of view, or resolution) alter the linear gradients, FRONSAC gradients provide marked improvements in experimental images without requiring alteration. This means that the required field mapping can be performed for a single waveform that can then be used to improve a wide range of scans, which is a critical feature for clinical application. Note R = 6 in this figure is an approximation signifying 12/64, 23/128, and 45/256 measurement lines. Black space in the images has been cropped for better visualization.

Figure 4 shows that once a FRONSAC NLG waveform has been well characterized by spatial and temporal mapping, it can be applied to a variety of desired Cartesian scan prescriptions and still produce profound improvements in undersampling artifacts. The columns of Fig. 4 show that adding the same FRONSAC waveform at different imaging slice orientations, or different sized fields of view (FOV), or different resolution improves undersampling artifacts in all cases. At higher resolution, there is effectively less relative FRONSAC encoding (fewer modulations of the encoding distribution per distance in k-space), so the improvement is somewhat reduced, but still easily observed. Importantly, the FRONSAC gradient never degrades image quality. Similarly, switching the readout line direction and thereby the undersampling direction changes the orientation of the undersampling artifacts, but does not diminish the effectiveness of the FRONSAC waveform.

### Compatibility with other contrast and acceleration strategies

**Figure 5:**
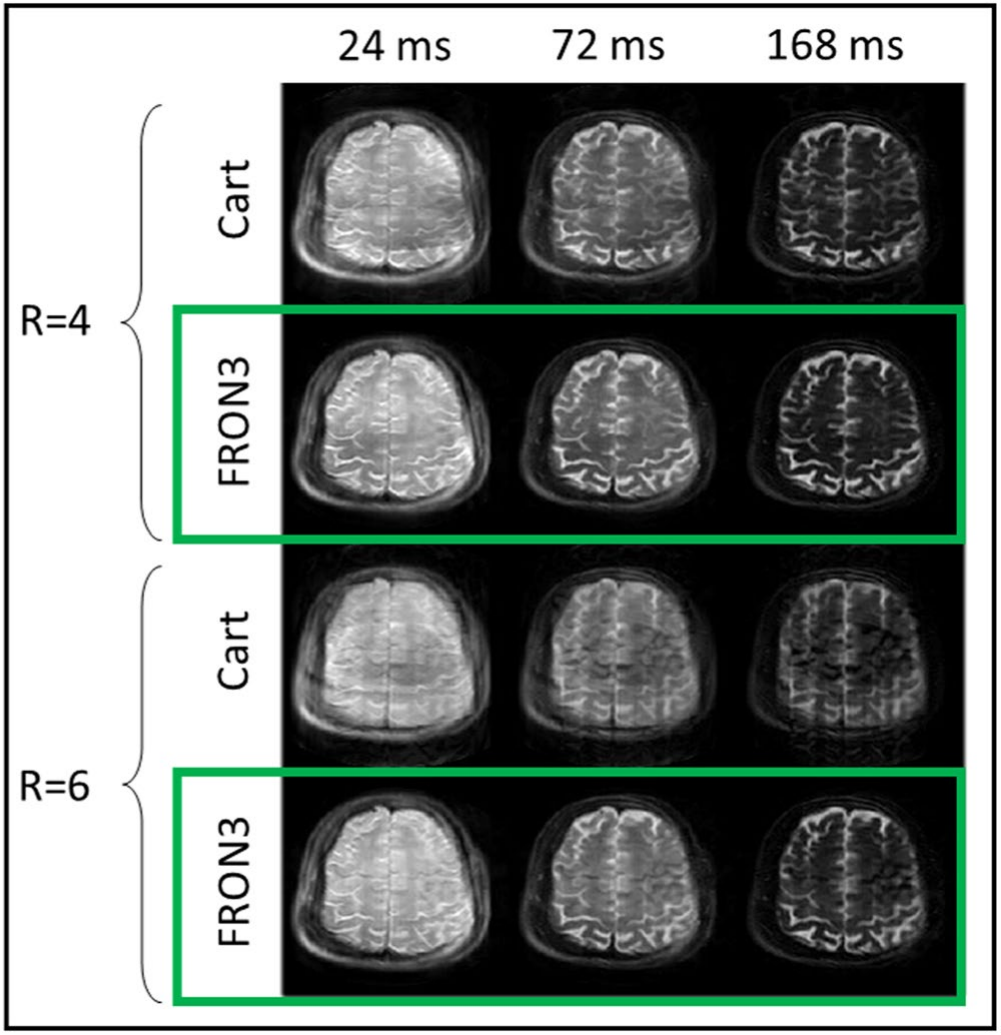
FRONSAC is compatible with other imaging methods. FRONSAC is applied to a turbo spin echo sequence with an echo train length (ETL) of 8 and effective echo times shown above each column. To calculate the actual scan time reductions the ETL is multiplied with the undersampling factor, yielding scan accelerations of 32 and 48 for these images. Experimental FRONSAC images maintain identical contrast to the Cartesian images at each effective echo time. Black space in the images has been cropped for better visualization.

Figure 5 demonstrates that FRONSAC is synergistic with other acceleration strategies such as turbo spin echo (TSE) imaging and FRONSAC gradients do not interfere with the standard contrast. In TSE imaging, the measurement time is reduced by acquiring a train of measurement lines in k-space instead of a single k-space line per repetition. Acceleration is proportional to the acquired number of k-space lines in the train or echo train length (ETL). TSE can be further accelerated by skipping k-space measurement lines, yielding undersampled TSE datasets like those shown in Fig. 5. The columns of Fig. 5 show that FRONSAC maintains identical contrast to the Cartesian TSE at each effective echo time while the rows with undersampling factor 4 and 6 show that FRONSAC reduces the undersampling artifacts.

### Compatibility with advanced reconstruction strategies

**Figure 6:**
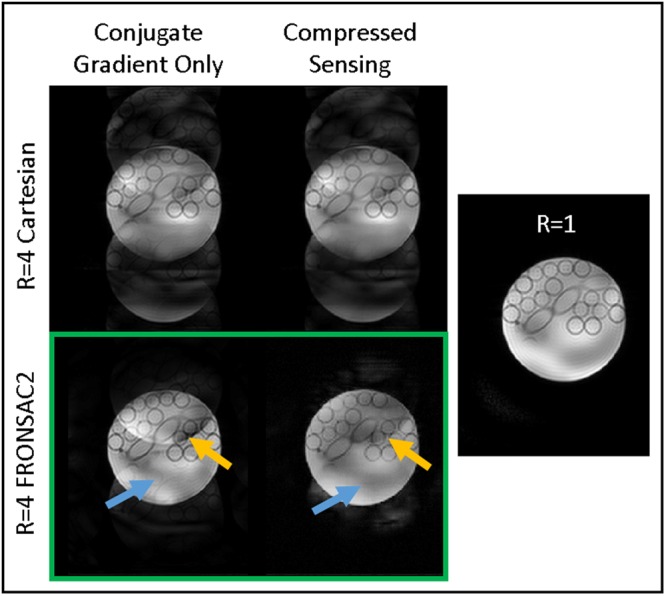
Images are reconstructed with conjugate gradient or with additional compressed sensing. Comparison of transform point spread functions have already indicated that NLG and FRONSAC encoding improve compatibility with compressed sensing^22^, and here that is demonstrated experimentally. Compressed sensing does not help remove artifacts from the Cartesian case, but when applied to FRONSAC2 image reconstruction it further removes ghosting artifacts such as the tubes (blue arrow) and edge of the phantom (orange arrow).

Recently, tremendous improvements in undersampled images have been found with advanced reconstruction methods such as compressed sensing, which promotes sparsity in an appropriate transformation domain^25,26^. It has previously been shown that NLG encoding, and FRONSAC in particular, can result in data that is better conditioned for reconstruction by compressed sensing^30,31^. Figure 6 shows that images reconstructed from accelerated FRONSAC2 data benefit greatly from a compressed sensing approach, while virtually no benefit is seen for images reconstructed with evenly undersampled Cartesian data without NLG encoding. These reconstructions further suggest that significant improvements can be made even with lower amplitude FRONSAC encoding with judicious choice of reconstruction algorithm. Details of the image reconstruction strategy is available in Supplementary Note S2.

### Applicability on different hardware configurations

**Figure 7:**
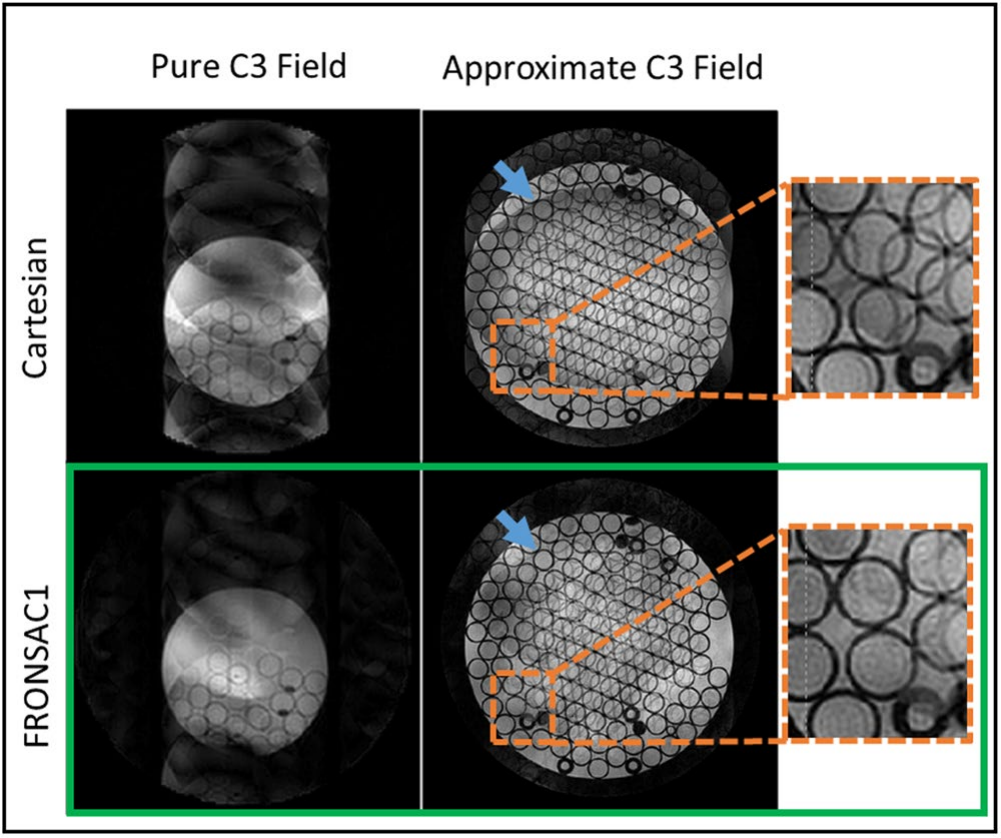
Imaging experiments are conducted at two research sites. Phantom images are reconstructed at R = 6. Only one nonlinear gradient waveform is applied with a C3 shape in the Yale MRRC images and an approximate C3 shape in the Freiburg images. At both research sites, FRONSAC reduces the undersampling artifacts. The insets show that the FRONSAC images produce less ghosting artifacts inside the tubes and at the edge of the phantom (blue arrow).

Finally, to illustrate the flexibility of this approach, data is presented in Fig. 7 from an entirely different set of hardware sited at University of Freiburg. This laboratory has built an 84-channel matrix gradient coil which is capable of generating a number of nearly arbitrary gradient shapes^32,33^. For this study, the 84 channels were run in a combination that approximates a C3 shape, and amplitude was then modulated sinusoidally in time. Due to the limited amplitude and single field shape used in that experiment, artifact reduction is somewhat lower than that shown in preceding figures. However, the reduction in undersampling artifacts compared to the Cartesian image is unmistakable, and more importantly it is similar to comparable C3 FRONSAC1 images acquired at Yale. This demonstrates, though improvements may be reduced by weaker field amplitude and fewer NLG channels, field purity does not significantly affect the FRONSAC experiment.

## Discussion

Adding FRONSAC encoding to linear undersampled trajectories improves imaging because the NLGs spread the sampling distribution in k-space, providing more information about the nominally “skipped” regions in k-space (Fig. S1). More precisely, the product of each NLG encoding with the receiver array encoding generates as many sampling distributions as coils^23^, and these independent distributions sample the gaps in the nominal trajectory (Fig. 1c,d). Since each stamp no longer unambiguously measures a point in k-space, it is necessary to measure these distributions for many overlapping positions and orientations, so the underlying k-space can be accurately deduced. This is the rationale for the FRONSAC waveform. The orientation of the distribution is modulated by the out of phase, rapidly oscillating timecourse of various NLG waveforms, while changes in position accumulate from evolution under the linear gradient. By extracting more spatial information from a smaller number of k-space lines, FRONSAC reduces the required scan time while still resulting in good image quality.

While FRONSAC gradients are extremely beneficial even when they are not optimally tailored to the scan prescription (Fig. 4) with regard to amplitude and frequency^22^, number of waveforms (Fig. 2), or field shape (Fig. 7), the optimal FRONSAC gradient for a given scan prescription can be qualitatively described by considering the distributions in k-space. The area of the distribution is proportional to the cumulative area under a FRONSAC waveform or its moment and should be large enough to spread the sampling distribution into the gaps of k-space created by undersampling. Therefore, higher amplitude gradients are better for larger undersampling factors^33^. Meanwhile the number of overlapping orientations of each sampling distribution, related to the frequency of the out of phase FRONSAC waveforms, should be comparable to the number of unknowns being sampled by each distribution, making higher frequencies more favorable. In experiments, the FRONSAC waveform amplitude and oscillation frequency must be kept within hardware and physiological limitations. As frequency increases, amplitude and slew rate would need to increase quadratically to maintain a given NLG moment. With very large NLG moments, the resolution needs to be adjusted to account for intravoxel dephasing. While the choice of NLG fields for this work was dictated by existing NLG hardware, it is possible to systematically optimize the NLG field shapes for specific applications and receive coil arrays^33^.

The particular tradeoffs and bandwidth limitations are highly hardware dependent. For example, experiments with the Freiburg 84 channel matrix coil, which was not optimized for FRONSAC encoding, used 70% of the maximum available gradient strength and could not be safely increased further for higher frequency acquisitions due to heating, whereas those on the Yale spherical harmonic coil used only 10% and showed no detectable heating. The slew rate limits of the Yale hardware, which was not designed for FRONSAC encoding, necessitate lower bandwidths for FRONSAC imaging, but even the current parameters yield significant improvements. As first generation custom hardware, it is difficult to specify what parameters can be expected in a clinical implementation. However, the presented results do demonstrate that widely achievable FRONSAC gradients, which do not violate *d****g****(t)/dt* limits of hardware or *d****B****(t)/dt* limits of peripheral nerve stimulation, can dramatically improve image quality without particular optimization, demonstrating the versatility of the method.

Many non-Cartesian trajectories can also improve parallel imaging without NLGs or additional hardware, yet the presented work is highly significant for several reasons. Notably, though high performance non-Cartesian trajectories have been well understood for many decades (spiral, rosette, radial), they are still in limited use for clinical applications^34–36^. One reason is that these methods can be highly sensitive to errors in the gradient trajectory, off-resonance effects, or subject motion, and another is that they can yield complicated contrast^34,37^. Cartesian FRONSAC, by contrast, is a small modification to the workhorse Cartesian sequence, so it shares many of the desirable characteristics of Cartesian imaging.

Resilience to errors in gradient trajectory as demonstrated in Fig. 3 is a particularly important feature of FRONSAC because it is directly linked with the ability to change image geometry on the fly, a vital requirement for clinical imaging. In other non-Cartesian trajectories, the gradient waveforms must change for scans of different resolution, FOV, or orientation, but without advanced equipment, it is infeasible to empirically field map the real output of each waveform^33,38^. Therefore, there is often a mismatch between the prescribed and executed waveforms, which can lead to serious degradations in image quality. In contrast, if such mismatches arise in Cartesian or Cartesian-FRONSAC imaging, the resulting artifacts are mild or even undetectable. Similarly, off-resonance effects and susceptibility gradients can have complicated and profound impacts on non-Cartesian images, but they result in simple localized artifacts in both Cartesian and Cartesian-FRONSAC imaging.

Like other non-Cartesian trajectories with a dynamic readout gradient, the FRONSAC waveform does require empirical mapping. However, unlike most non-Cartesian trajectories, once this single mapping is performed, the same waveform can be used for a huge range of scan prescriptions, as shown in Fig. 4, and still yield significant image improvement. Some image quality improvement is expected when extending this work to any imaging orientation, and has been shown for oblique orientations, but the extent may vary.

A final important strength of Cartesian-FRONSAC, compared to many non-Cartesian trajectories, is that it also preserves simplified contrast behavior, for example in multiecho acquisitions^39^. Cartesian-FRONSAC preserves the blockwise sampling of central k-space in sequences such as echo planar imaging or turbo spin echo, which can further compound image acceleration. Our results in Fig. 5 show that controlling contrast in FRONSAC TSE is straightforward, and images are indistinguishable from the Cartesian case, except for better undersampling behavior.

Several non-Cartesian trajectories that deserve special mention are bunched phase encoding, zigzag sampling and wave-CAIPI techniques, which bear some resemblance to the FRONSAC approach^40–42^. These techniques use standard linear gradients, typically with far fewer oscillations per readout, and thus are a modified k-space trajectories which inherently collect points in k-space. In contrast, FRONSAC uses NLG fields, which induce spatially-varying encoding and cause the k-space sampling distribution to change during the acquisition. Additionally, the above mentioned techniques are often limited by peripheral nerve stimulation from the rapidly oscillating linear gradients, whereas the NLGs used in FRONSAC switch multipolar fields, which mitigate peripheral nerve stimulation^8^. And like other non-Cartesian trajectories, the gradient waveforms used in these techniques must be adapted to scan geometry, introducing potential challenges in field mapping.

Importantly, however, wave-CAIPI and other non-Cartesian trajectories should not be regarded as competitive with FRONSAC encoding but rather synergistic. FRONSAC preserves the features of the underlying trajectory while allowing larger gaps in k-space. As the challenges of these non-Cartesian trajectories are overcome, each method can be still further accelerated by allowing larger gaps in k-space and using FRONSAC to better encode these gaps. Previously published simulations show that FRONSAC encoding can improve nearly any trajectory, whether Cartesian or not^33^.

Finally, FRONSAC encoding has some advantages in reconstruction that are likely to enhance clinical applicability. Because of the highly parallelizable nature of the reconstruction, scan quality can be verified in ~15 seconds with two conjugate gradient iterations using just one GPU, and it is reasonable to predict that a near instantaneous image could be generated from an inverse transformation found via machine learning^43^. Furthermore, the diffuse FRONSAC encoding in k-space creates unstructured undersampling artifacts, which are suitable for suppression with compressed sensing (Fig. 6). Compressed sensing improves other trajectories, including many non-Cartesian or randomly sampled acquisitions, but the addition of FRONSAC gradients improves compressed sensing reconstructions without requiring alteration of the underlying linear trajectory.

In summary, the presented work not only proves the experimental *feasibility* of Cartesian-FRONSAC, but also hypothesizes and verifies features that establish the experimental *practicality* of Cartesian-FRONSAC. The method is robust to the most inescapable hardware errors, such as small timing errors or off-resonance spins. It requires minimal field mapping to improve a wide range of scan prescriptions, whether using a different contrast or a different scan geometry. The nonlinear fields themselves do not require a high degree of purity and can be realized from different hardware configurations. Finally, the method is fully compatible with other acceleration strategies, such as multi-echo acquisition or compressed sensing reconstruction. This suggests that Cartesian-FRONSAC is both an effective and highly practical approach to improving scan acceleration in a broad range of clinical applications.

## Methods

### Hardware

All imaging experiments were performed on a 3T MRI scanner (MAGNETOM Trio Tim, Siemens Healthcare, Erlangen, Germany). Parallel data acquisition was performed using an integrated 8 channel RF head coil (Siemens). The majority of the phantom and *in vivo* experiments were performed at the Yale Magnetic Resonance Research Center using a NLG insert (Tesla Engineering Ltd, Storrington, UK) rated at 321 A with an inner diameter of 380 mm which generates 3 spherical harmonic gradient fields: x^3^ − 3xy^2^, 3yx^2^ − y^3^ and x^2^ + y^2^ (commonly known as C3, S3, and Z2) (Fig. 1b). The gradient coil is capable of achieving maximum C3, S3, and Z2 fields of 3254.8 mT/m^3^, 3155.4 mT/m^3^ and 475.08 mT/m^2^. Phantom experiments were also performed at the University Medical Center Freiburg using an 84 channel matrix gradient coil driven by 12 gradient amplifiers rated at 150 amps with an inner diameter of 350 mm which allows flexible gradient field shapes generation^33^. A cluster of elements capable of achieving NLG fields approximating the C3 spherical harmonic field was used. The approximate maximum C3 strength of the matrix gradient is 452 mT/m^3^. The cluster was set up for general scanning and not optimized for generating the C3 shape.

### Imaging experiments

For all experiments, unless otherwise stated in the figures, the field of view was 250 mm × 250 mm of a transverse slice at isocenter acquired with 1024 samples per readout line with 128 lines. Linear gradient strengths are set according to typical Nyquist prescriptions for Cartesian imaging for a 128^2^ matrix. FRONSAC NLG waveforms were added to Cartesian gradient echo (FLASH) and turbo spin echo sequences as detailed in Supporting Background Information. Temporally, the “C3” and “Z2” fields follow a sine waveform while the “S3” gradient follows a cosine waveform.

Sequence parameters for phantom and *in vivo* gradient echo (FLASH) imaging performed at Yale University were as follows: TR = 1000 ms/TR = 600 ms (respectively); TE = 18 ms; bandwidth = 50 Hz/pixel; flip angle = 30°/15° (respectively); slice thickness = 3 mm, acquisition matrix = 128 × 1024, maximum C3/S3/Z2 strength = 325.3 mT/m^3^, 316.7 mT/m^3^ and 41.6 mT/m^2^ with oscillation frequency of ${w}_{0}$/2pi = 3.2 kHz.

Sequence parameters for *in vivo* turbo spin echo imaging performed at Yale University were: TR = 3000 ms; turbo factor = 8; echo spacing = 24 ms; bandwidth = 100 Hz/pixel; slice thickness = 5 mm, acquisition matrix = 128 × 1024, maximum C3/S3/Z2 strength = 390.7 mT/m^3^, 380.1 mT/m^3^ and 50.0 mT/m^2^ with oscillation frequency of ${w}_{0}$/2pi = 4.8 kHz. Contrast at different effective echo times is selected by changing the acquisition order of the k-space lines.

Sequence parameters for phantom gradient echo imaging performed at the University of Freiburg Medical Center were: TR = 700 ms; TE = 11.2 ms; bandwidth = 78.125 Hz/pixel; flip angle = 20°; slice thickness = 5 mm, acquisition matrix = 256 × 1024, maximum C3 strength = 293.9 mT/m^3^ with oscillation frequency of ${w}_{0}$/2pi = 5 kHz.

In the Freiburg University experiments, sequence programing was performed with the open-source environment Pulseq^44^. The NLG trajectories were measured with a 4 channel field camera. The field camera measurements were not used for spatial gradient field mapping but rather for temporal trajectory mapping of the gradient waveforms. The field camera measurements were used for finding frequency and phase information of the NLG oscillations as well as systematic gradient delays. Although delays can be corrected in post processing, in this work we chose to account for the delays in the pulse sequence by adjusting the starting time of the gradient waveforms.

In the Yale University experiments, the NLG trajectories were measured with a phase mapping sequence as described by Wang *et al*.^33^. A 6th order polynomial fit was used to fit the spatial information of the NLG evolution over time, which was then used to model field evolution over the entire scan area. Coil array sensitivity maps were acquired in a separate scan.

The Human Investigation Committee granted Institutional Review Board approval to image healthy human volunteers. After obtaining informed consent the brains of two volunteers were imaged. Subjects reported no discomfort during the scans. The study was in accordance with the Declaration of Helsinki.

Nyquist sampling rate determined the number of lines of data acquired to avoid image aliasing. Each line of data was oversampled. Note that oversampling each line does not increase the scan time.

### Image reconstruction

Undersampling was performed after acquisition during image reconstruction by discarding lines of data. GRAPPA reconstructions incorporate additional auto calibration signal (ACS) lines from the center of k-space and employed a 4 × 5 kernel. Coil sensitivity maps are masked to the sensitive region of the receive coil array. All calculations were performed in MATLAB (MathWorks Inc, Natick, Massachusetts, USA). All reconstructions were performed via a conjugate gradient algorithm with 10 iterations using the GPU. Additionally, some datasets were processed with a conjugate gradient compressed sensing algorithm using an l1 norm minimization in the sparse wavelet domain as detailed in Supporting Background Information^25^. A total variation constraint was used in the compressed sensing algorithm with sparsifying transform chosen to be Daubechies wavelets with 4 vanishing moments and with 6 levels of wavelet decomposition. The wavelet transform penalty was 0.001 and the total variation constraint was 0.01. The algorithm ran through 15 conjugate gradient iterations. Reconstructions were performed on a 64-bit Linux workstation (Intel® Xeon w5580, 8 processors at 3.2 GHz, 32 core, 48 GiB RAM) with one GeForce GTX 1080 GPU (Nvidia®) and on a 64-bit Linux workstation (Intel® Xeon x5680, 11 processors at 3.33 GHz, 66 core, 94.5 GiB RAM) with two GeForce GTX 1080 GPUs (Nvidia®). Fully sampled conjugate gradient recons required ~14 minutes of reconstruction time, while 6-fold undersampled scans required ~1.5 minutes in reconstruction time. Data sets process with compressed sensing require ~35 minutes of reconstruction time.

## Electronic supplementary material

Supplementary Video S1

Supplementary Video S2

Supplementary Video S3

Supplementary Video S4

Supplementary Video S5

Supplementary Information

## Data Availability

The datasets and materials generated during the current study are available from the corresponding author on reasonable request.
