# Supplementary material for: Clinical Potential of a New Approach to MRI Acceleration
Source: Sci Rep. 2019 Feb 13;9:1912. doi: 10.1038/s41598-018-36802-5 (PMC6374397; doi:10.1038/s41598-018-36802-5)
Supplement: Supplementary file 6 — Supplementary Information [file 41598_2018_36802_MOESM6_ESM.docx]

**Supplementary Information**

**Clinical Potential of a New Approach to MRI Acceleration**

Nadine L. Dispenza^1^, Sebastian Littin^2^, Maxim Zaitsev^2^, R. Todd Constable^3,4^, Gigi Galiana^3*^

^1^Department of Biomedical Engineering, Yale University, New Haven, CT, USA

^2^Department of Diagnostic Radiology, Medical Physics, University Medical Center Freiburg, Breisacher Str. 60a, 79106, Freiburg, Germany

^3^Department of Radiology and Biomedical Imaging, Yale University, New Haven, CT 06520, USA

^4^Department of Neurosurgery, Yale University, New Haven, CT 06520, USA

Correspondence should be addressed to G.G. ([gigi.galiana@yale.edu](mailto:gigi.galiana@yale.edu))

**Supplementary Note S1**

**FRONSAC encoding with nonlinear gradients.** In the FRONSAC method, modest oscillating nonlinear gradients fields x^3^-3xy^2^, 3yx^2^-y^3^ and x^2^+y^2^ with common names C3, S3 and Z2, respectively, are added to a linear encoding trajectory. The signal s_q_ from the q-th RF receive channel with sensitivity C_q_ from magnetization m(**x**) at location **x** experiencing an encoding phase Φ(**x**,t) in the region of interest Ω is:

$s_{q}(t)=\int_{\Omega} m(\boldsymbol{x})C_{q}(\boldsymbol{x})e^{i \Phi\left( \mathbf{x},t \right)}d\boldsymbol{x}$ (1)

The phase is generated by the evolution of the nonlinear gradients fields over time due to the gradient moments, **k**(t) = $[k_{x}\left( t \right), k_{y}\left( t \right), k_{x^{3}-3xy^{2}}\left( t \right), k_{{3yx}^{2}-y^{3}}, k_{x^{2}+y^{2}}\left( t \right)]$, applied to the nonlinear gradient fields **Ψ**(x) according to:

$\Phi\left( \mathbf{x},t \right)=\boldsymbol{k}^{T}(t)\boldsymbol{\Psi}(x)$ (2)

The gradient moment, **k** (t), describes the trajectory through k-space and is related to the waveform of the gradients, **g**(τ) =$[g_{x}\left( t \right), g_{y}\left( t \right), g_{x^{3}-3xy^{2}}\left( t \right), g_{{3yx}^{2}-y^{3}}, g_{x^{2}+y^{2}}\left( t \right)]$

via the gyromagnetic ratio, γ, as follows:

$\mathbf{k}(t)=\gamma\int_{0}^{t} \boldsymbol{g}(\tau)d\tau$ (3)

The gradients can be grouped by linear gradients g_L_(t) = [g_x_(t), g_y_(t)] and nonlinear gradients

g_NL_ = [${g_{x^{3}-3xy^{2}}\left( t \right), g}_{{3yx}^{2}-y^{3}},g_{x^{2}+y^{2}}\left( t \right)]. D$epending on gradient hardware capabilities the nonlinear gradient vector can contain an arbitrary number of nonlinear gradients. The linear gradients follow a Cartesian trajectory and the FRONSAC nonlinear gradients are played with amplitude, A_NL,_ and frequency, w_0,_ so that

$\boldsymbol{g}_{NL}\left( t \right)=[A_{NL}\sin\left( w_{0}t \right),A_{NL}\cos\left( w_{0}t \right), A_{NL}\sin\left( w_{0}t \right)]$ (4)

**Supplementary Note S2**

**Image reconstruction.** All gradient and coil encoding can be combined into an encoding matrix (E) and the signal equation can be simplified: s=Em where E is n_q_n_t_ by n_v_ where v is the number of voxels to reconstruct. Solving for $\hat{m}$ via pseudo inversion is too slow and memory intensive therefore a conjugate gradients technique is used to iterate towards $\hat{m}$. The conjugate gradients approach can process E row by row to reduce memory requirements and enable parallelization for GPU computations can be readily implemented. The number of iterations in the conjugate gradients approach must be adjusted to avoid bias from too few iterations and noise amplification from too many iterations. Typically, 10 iterations were found sufficient for this work. The compressed sensing reconstructions used an iterative conjugate gradient method seeks to minimize:

$m=\arg min\left\{ \left\| Em-s \right\|_{2}+\left\| \xi m \right\|_{1} \right\}$ (5)

Where $\xi$ denotes the sparsifying transorm. The inverse for the data consistency term is calculated with iterative GPU conjugate gradients reconstruction.


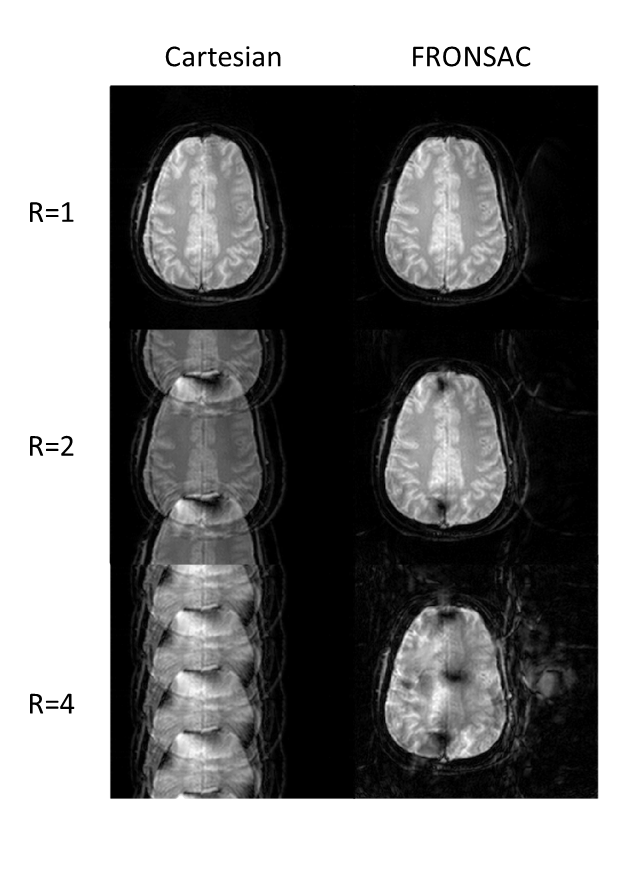


**Fig. S1:** Undersampled Cartesian-FRONSAC in-vivo datasets acquired with a single receive channel result in images that have less undersampling artifact than the Cartesian images. The diffuse FRONSAC encoding functions sample some information in the gaps of k-space, however some folded over pixels in the image cannot be resolved without additional coil information.


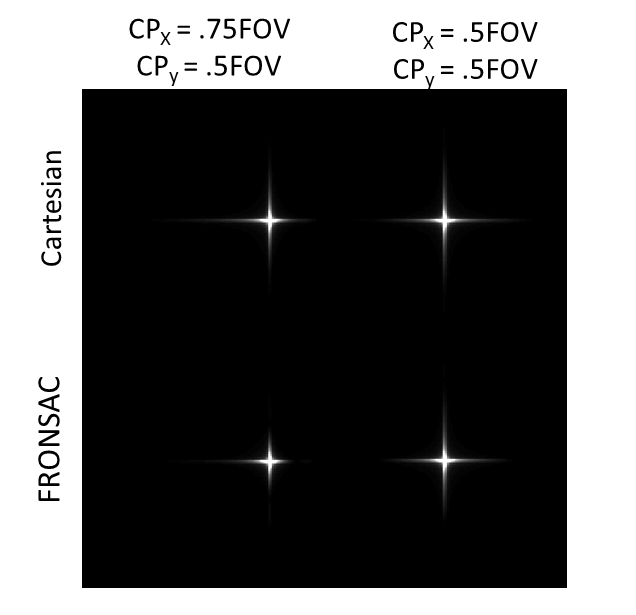


**Fig. S2:** FRONSAC, unlike other NLG methods, does not have significantly spatially varying resolution as demonstrated with these simulated PSFs at two different center placements (CPs) displayed on a linear scale. This is because only small nonlinear perturbations are added to the Cartesian sequence, which do not significantly change the extent of the k-space traversed. The overall extent of the local k-spaces is very similar such that the effective resolution of the FRONSAC images are almost equivalent to the resolution of the Cartesian images.
